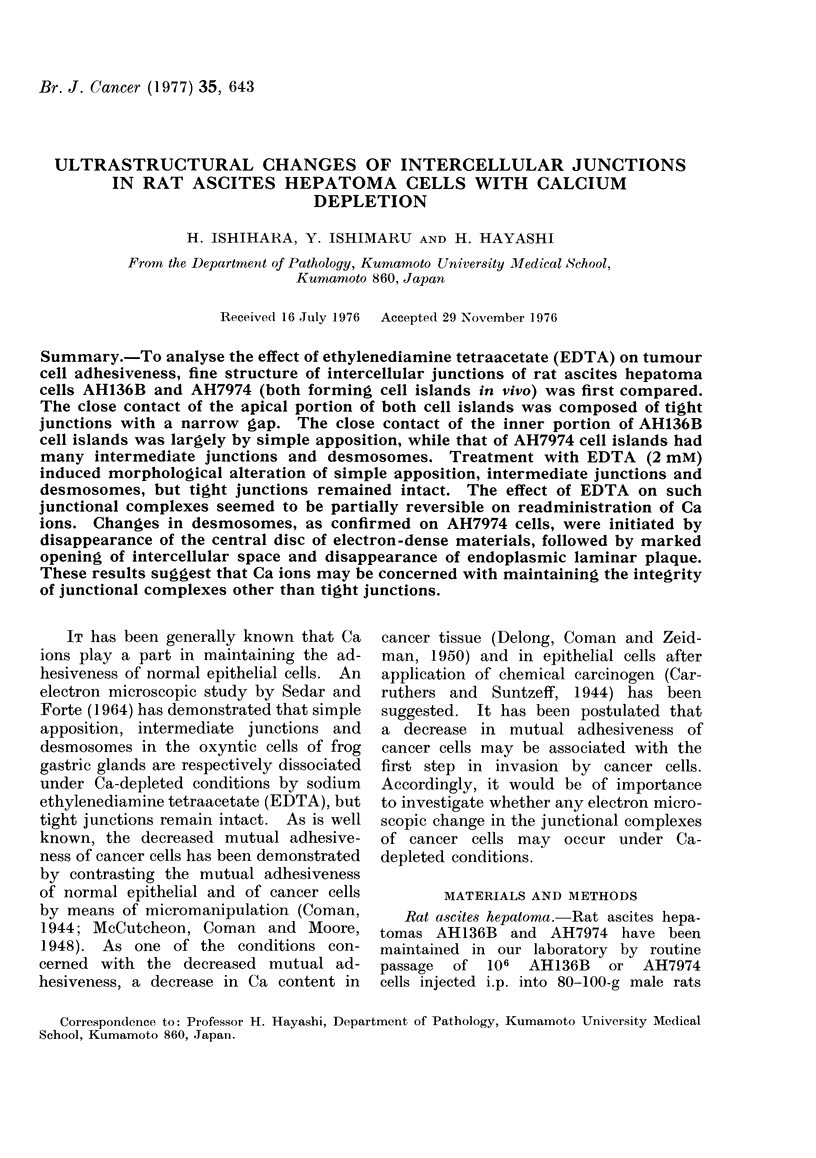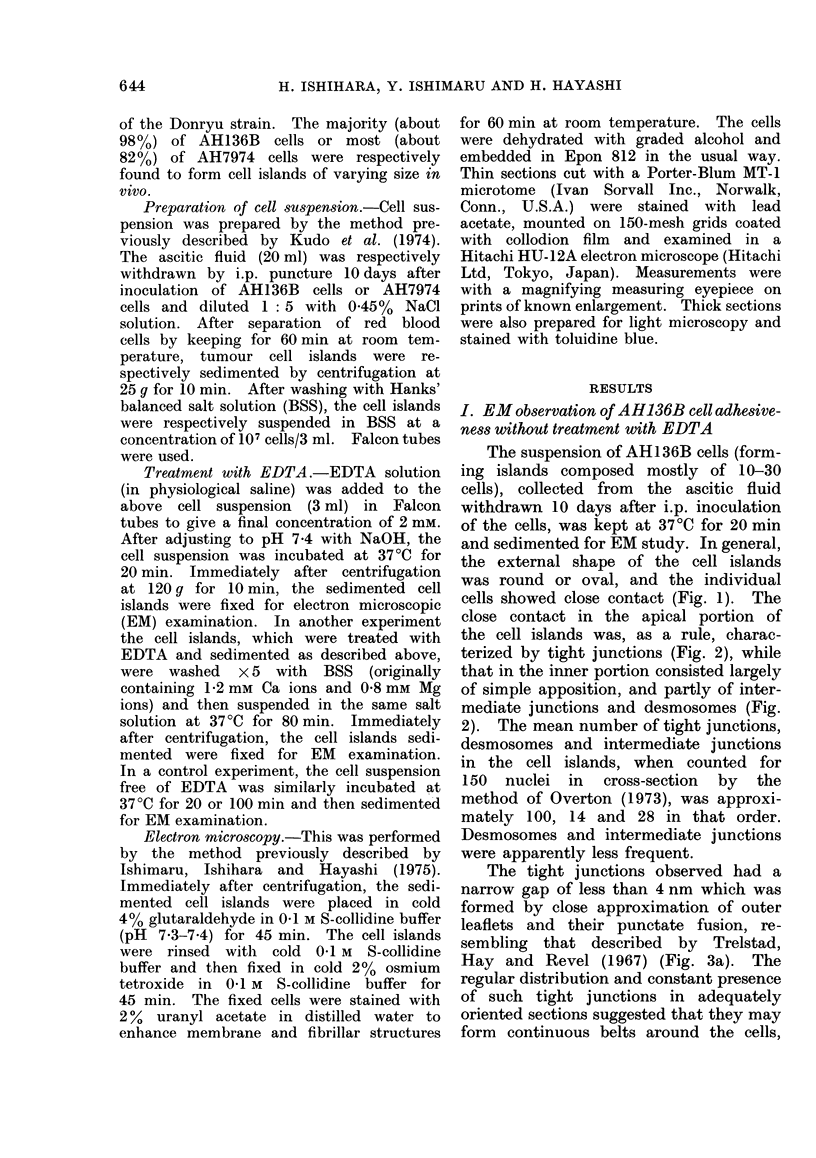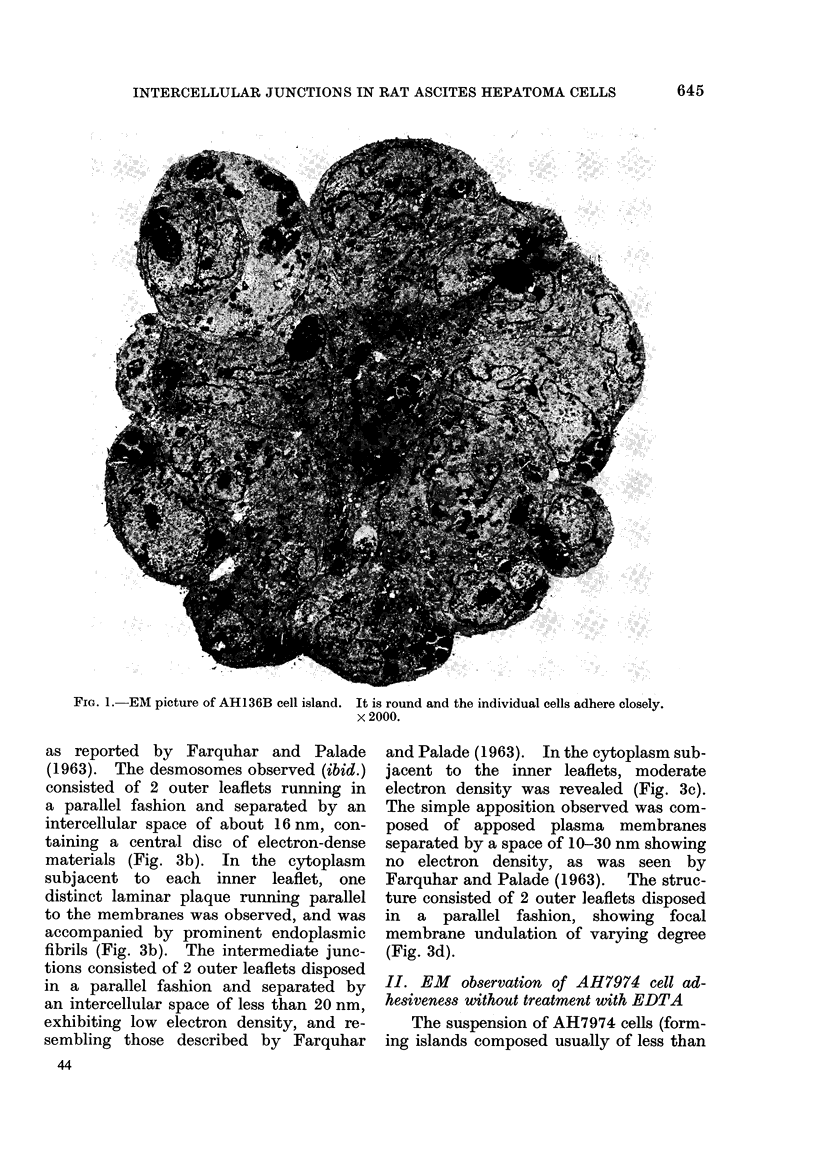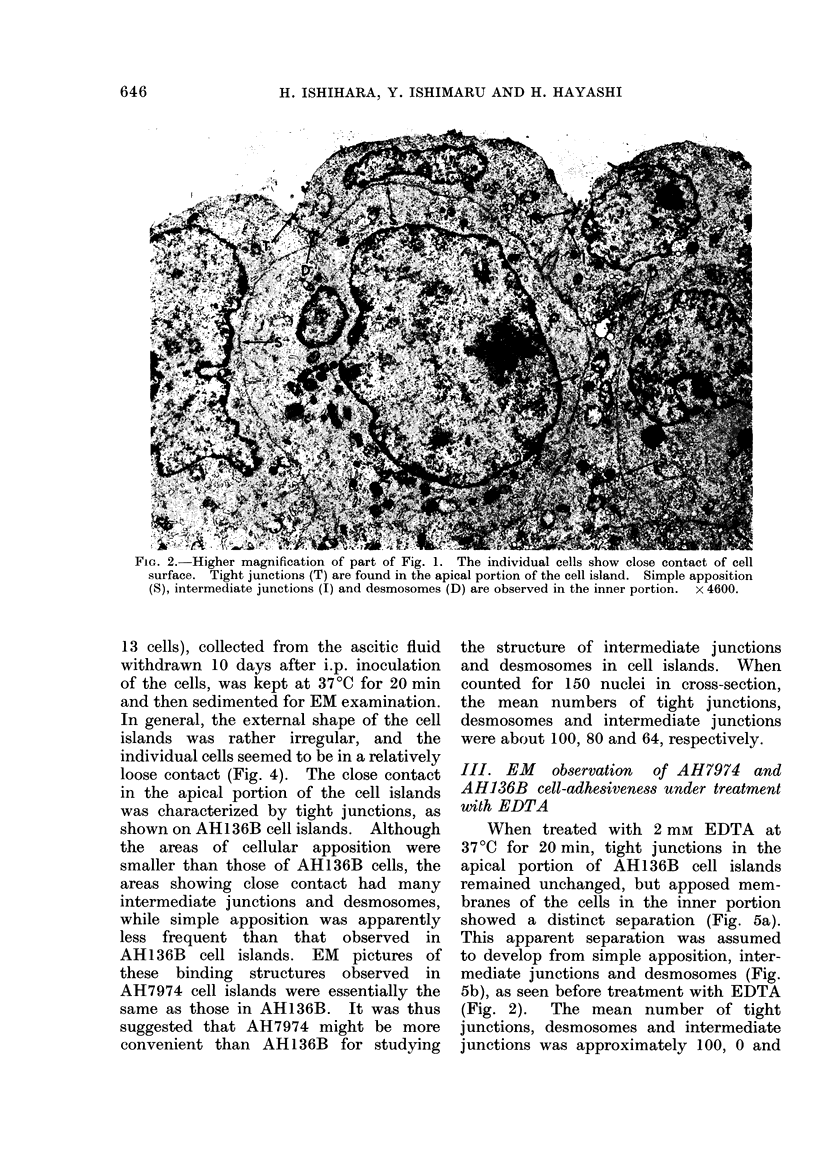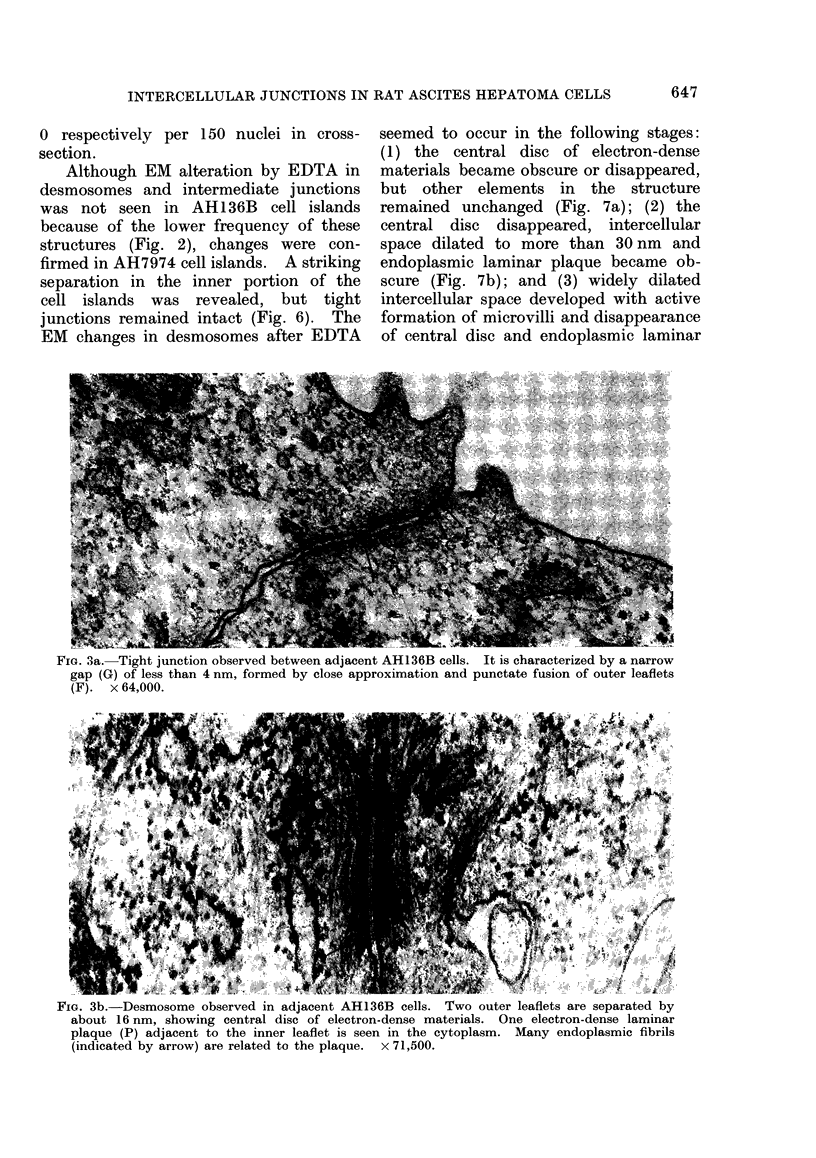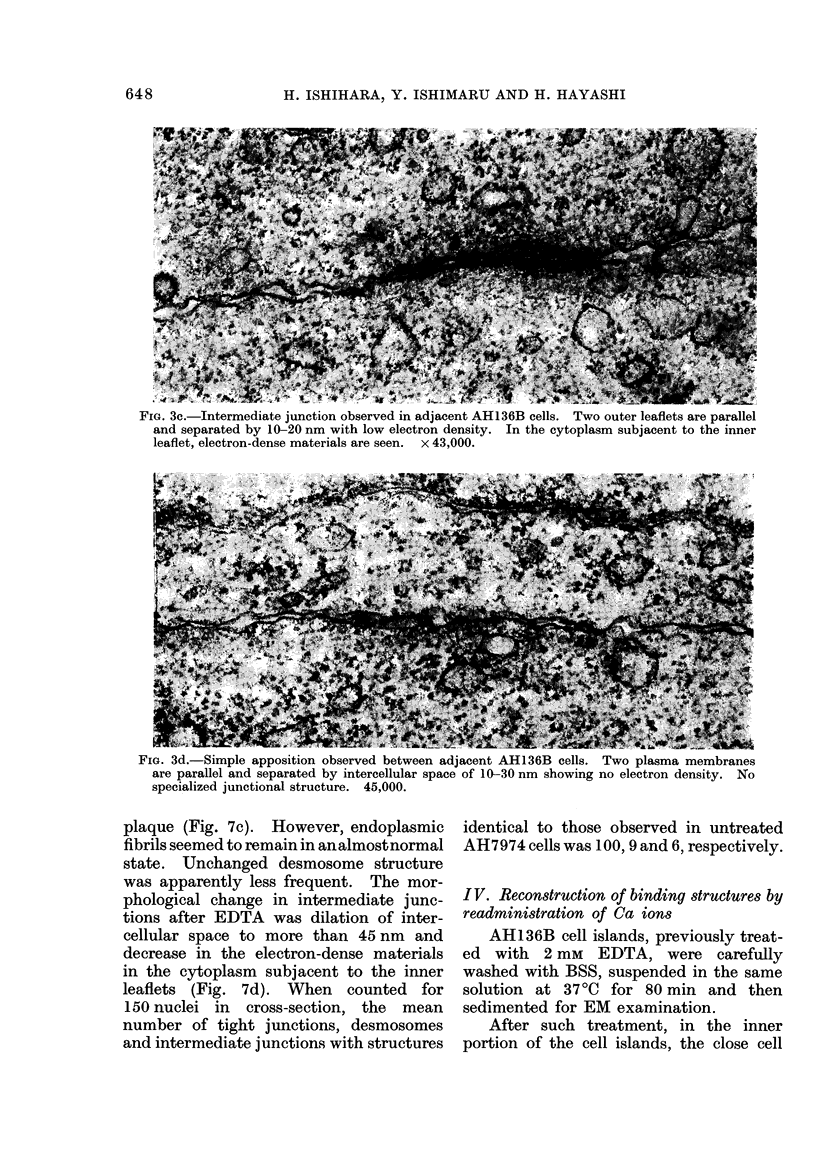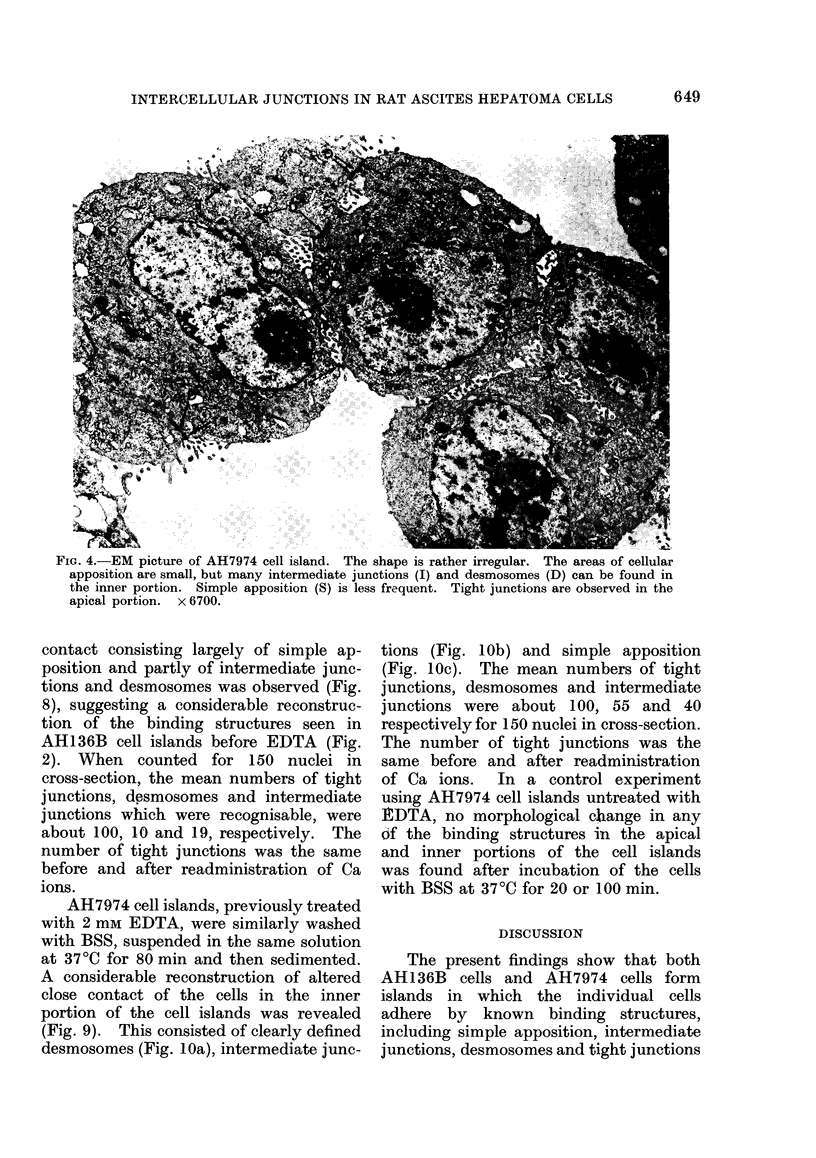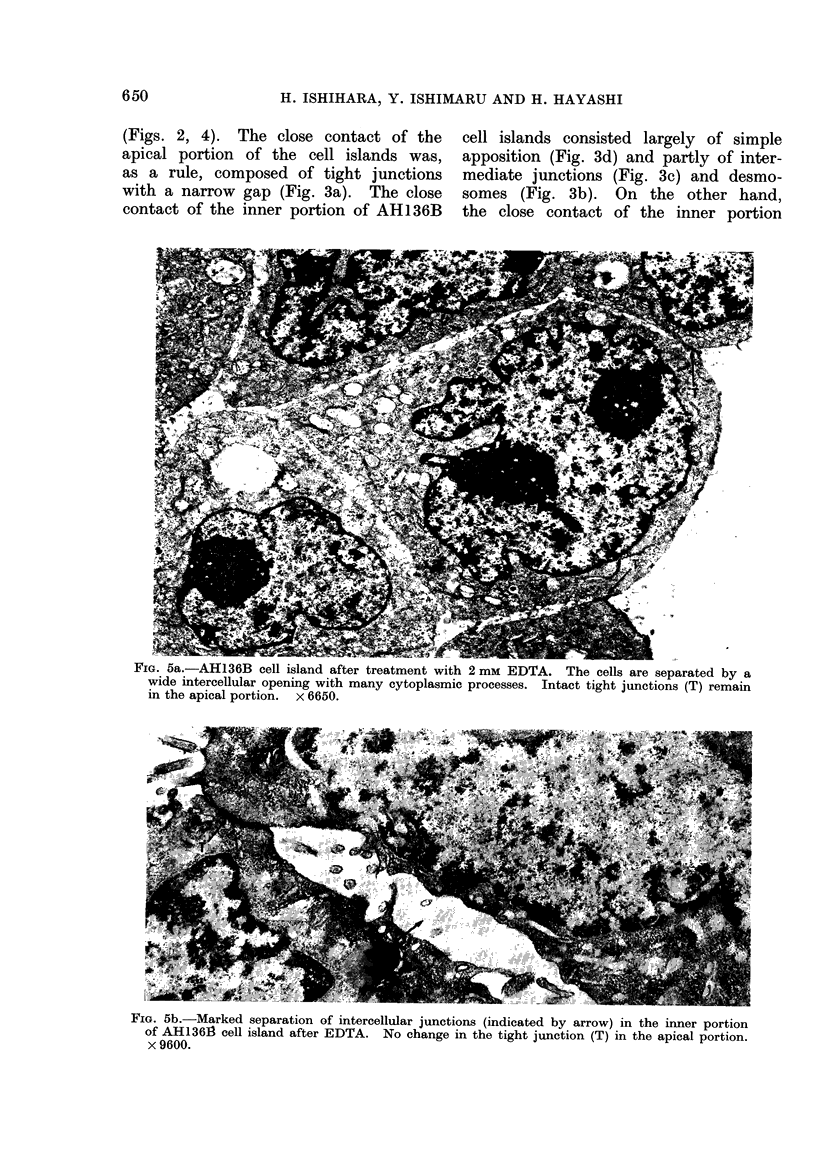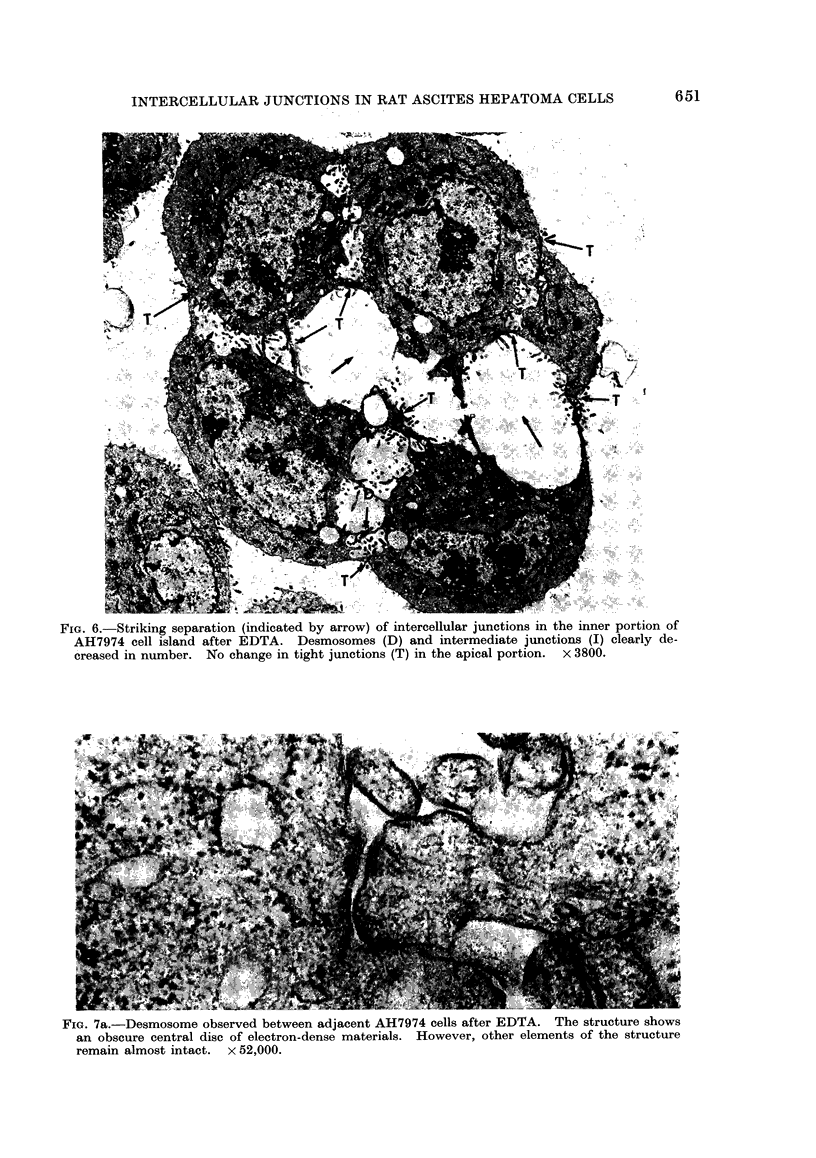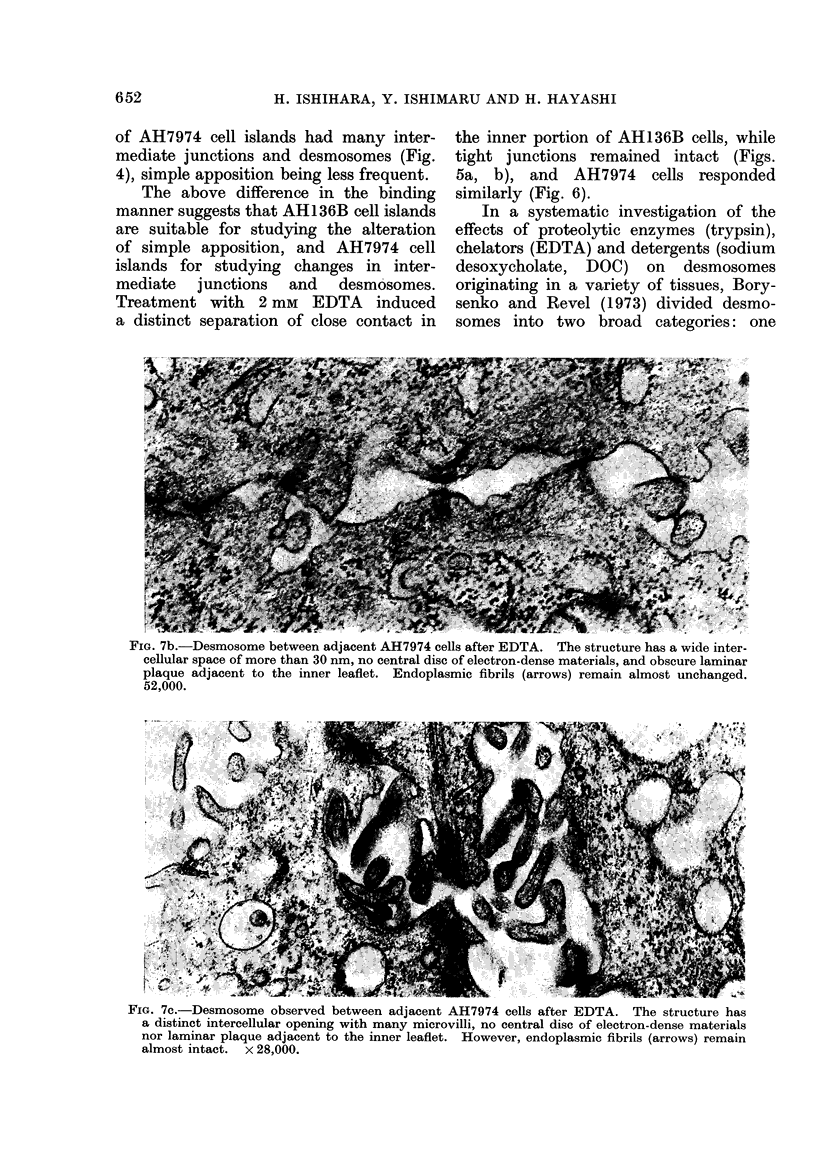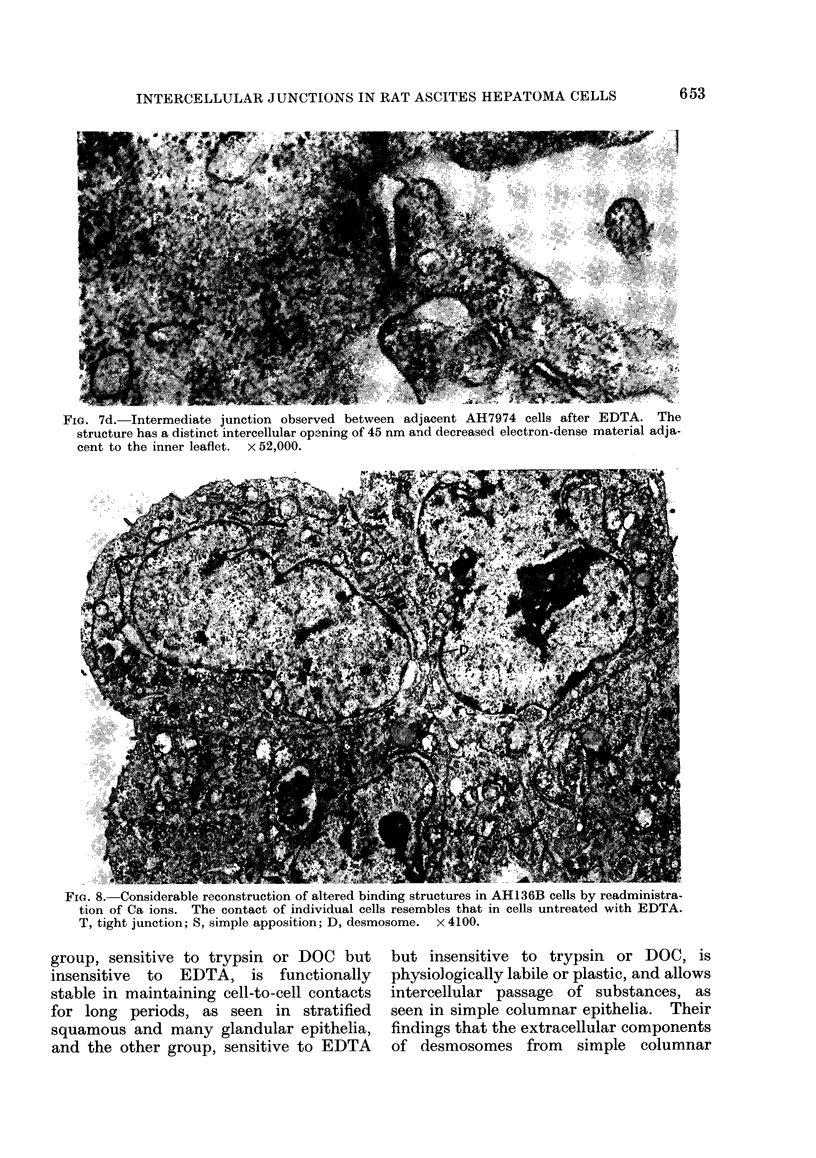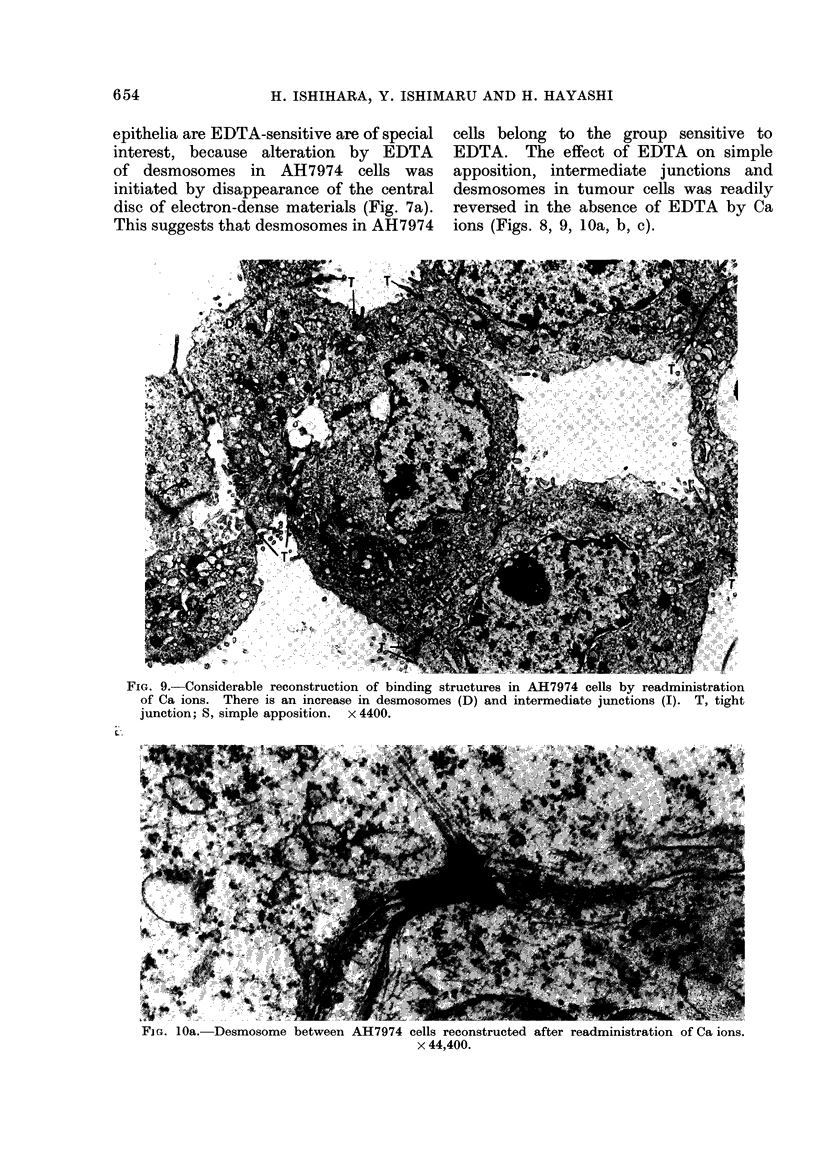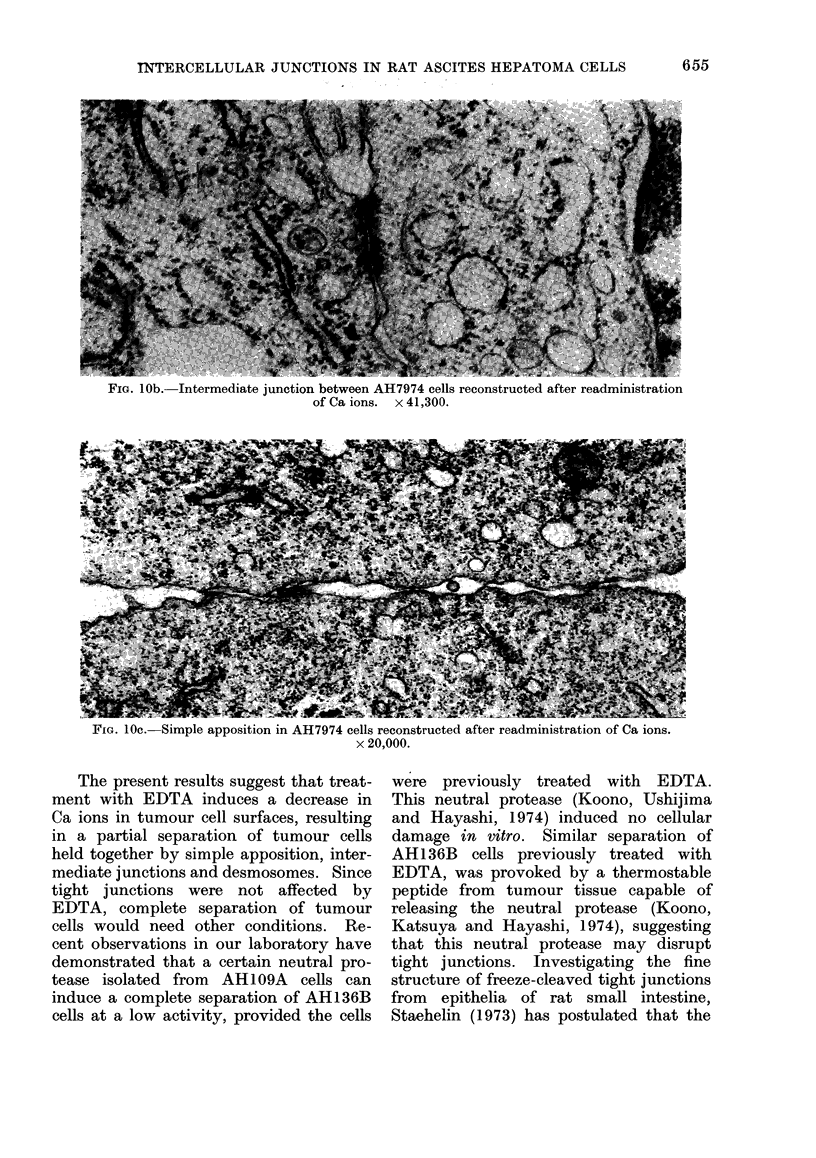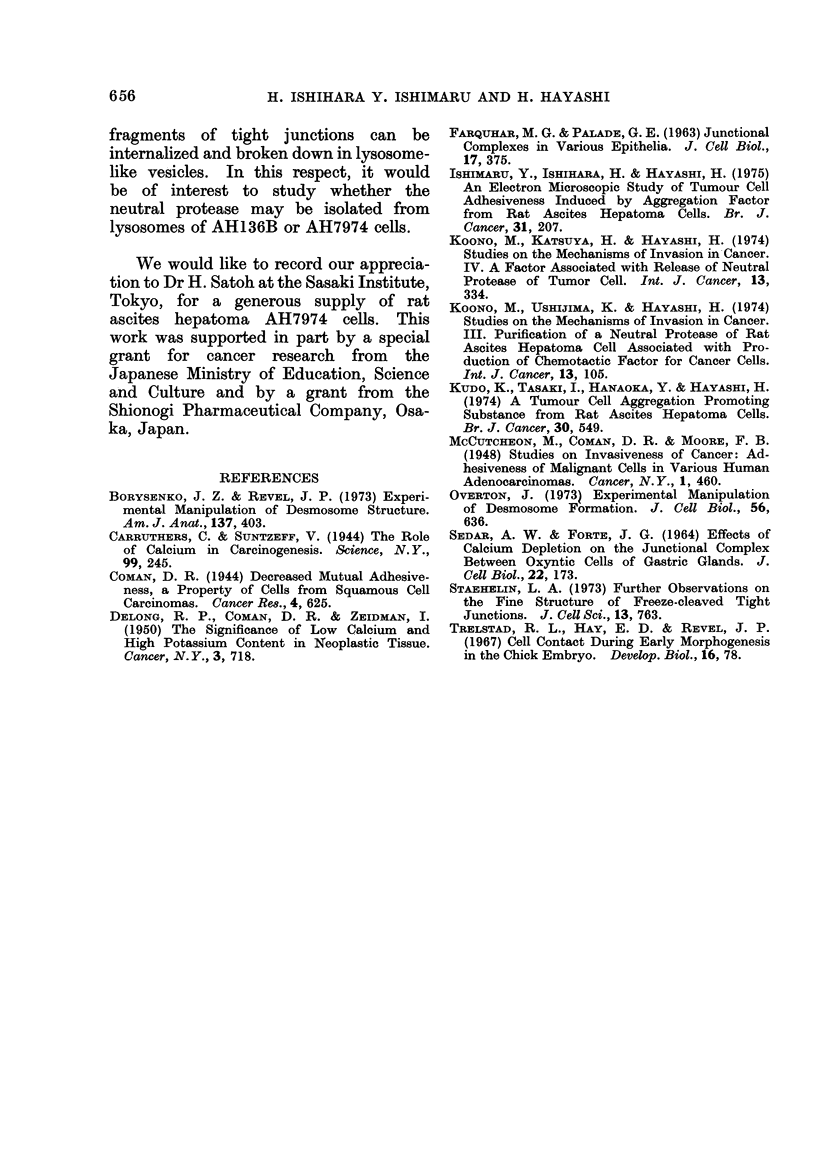# Ultrastructural changes of intercellular junctions in rat ascites hepatoma cells with calcium depletion.

**DOI:** 10.1038/bjc.1977.100

**Published:** 1977-05

**Authors:** H. Ishihara, Y. Ishimaru, H. Hayashi

## Abstract

**Images:**


*Br. J. Cancer* (1977) **35**, 643

### ULTRASTRUCTURAL CHANGES OF INTERCELLULAR JUNCTIONS IN RAT ASCITES HEPATOMA CELLS WITH CALCIUM DEPLETION

H. ISHIHARA, Y. ISHIMARU AND H. HAYASHI

*From the Department of Pathology, Kumamoto University Medical School, Kumamoto 860, Japan*

Received 16 July 1976 Accepted 29 November 1976

**Summary.—To analyse the effect of ethylenediamine tetraacetate (EDTA) on tumour cell adhesiveness, fine structure of intercellular junctions of rat ascites hepatoma cells AH136B and AH7974 (both forming cell islands *in vivo*) was first compared. The close contact of the apical portion of both cell islands was composed of tight junctions with a narrow gap. The close contact of the inner portion of AH136B cell islands was largely by simple apposition, while that of AH7974 cell islands had many intermediate junctions and desmosomes. Treatment with EDTA (2 mM) induced morphological alteration of simple apposition, intermediate junctions and desmosomes, but tight junctions remained intact. The effect of EDTA on such junctional complexes seemed to be partially reversible on readministration of Ca ions. Changes in desmosomes, as confirmed on AH7974 cells, were initiated by disappearance of the central disc of electron-dense materials, followed by marked opening of intercellular space and disappearance of endoplasmic laminar plaque. These results suggest that Ca ions may be concerned with maintaining the integrity of junctional complexes other than tight junctions.**

IT has been generally known that Ca ions play a part in maintaining the adhesiveness of normal epithelial cells. An electron microscopic study by Sedar and Forte (1964) has demonstrated that simple apposition, intermediate junctions and desmosomes in the oxyntic cells of frog gastric glands are respectively dissociated under Ca-depleted conditions by sodium ethylenediamine tetraacetate (EDTA), but tight junctions remain intact. As is well known, the decreased mutual adhesiveness of cancer cells has been demonstrated by contrasting the mutual adhesiveness of normal epithelial and of cancer cells by means of micromanipulation (Coman, 1944; McCutcheon, Coman and Moore, 1948). As one of the conditions concerned with the decreased mutual adhesiveness, a decrease in Ca content in cancer tissue (Delong, Coman and Zeidman, 1950) and in epithelial cells after application of chemical carcinogen (Carruthers and Suntzeff, 1944) has been suggested. It has been postulated that a decrease in mutual adhesiveness of cancer cells may be associated with the first step in invasion by cancer cells. Accordingly, it would be of importance to investigate whether any electron microscopic change in the junctional complexes of cancer cells may occur under Ca-depleted conditions.

### MATERIALS AND METHODS

*Rat ascites hepatoma.*—Rat ascites hepatomas AH136B and AH7974 have been maintained in our laboratory by routine passage of $10^6$ AH136B or AH7974 cells injected i.p. into 80–100-g male rats

Correspondence to: Professor H. Hayashi, Department of Pathology, Kumamoto University Medical School, Kumamoto 860, Japan.

of the Donryu strain. The majority (about 98%) of AH136B cells or most (about 82%) of AH7974 cells were respectively found to form cell islands of varying size *in vivo*.

*Preparation of cell suspension.*—Cell suspension was prepared by the method previously described by Kudo *et al.* (1974). The ascitic fluid (20 ml) was respectively withdrawn by i.p. puncture 10 days after inoculation of AH136B cells or AH7974 cells and diluted 1 : 5 with 0·45% NaCl solution. After separation of red blood cells by keeping for 60 min at room temperature, tumour cell islands were respectively sedimented by centrifugation at 25 *g* for 10 min. After washing with Hanks' balanced salt solution (BSS), the cell islands were respectively suspended in BSS at a concentration of $10^7$ cells/3 ml. Falcon tubes were used.

*Treatment with EDTA.*—EDTA solution (in physiological saline) was added to the above cell suspension (3 ml) in Falcon tubes to give a final concentration of 2 mM. After adjusting to pH 7·4 with NaOH, the cell suspension was incubated at 37°C for 20 min. Immediately after centrifugation at 120 *g* for 10 min, the sedimented cell islands were fixed for electron microscopic (EM) examination. In another experiment the cell islands, which were treated with EDTA and sedimented as described above, were washed ×5 with BSS (originally containing 1·2 mM Ca ions and 0·8 mM Mg ions) and then suspended in the same salt solution at 37°C for 80 min. Immediately after centrifugation, the cell islands sedimented were fixed for EM examination. In a control experiment, the cell suspension free of EDTA was similarly incubated at 37°C for 20 or 100 min and then sedimented for EM examination.

*Electron microscopy.*—This was performed by the method previously described by Ishimaru, Ishihara and Hayashi (1975). Immediately after centrifugation, the sedimented cell islands were placed in cold 4% glutaraldehyde in 0·1 M S-collidine buffer (pH 7·3–7·4) for 45 min. The cell islands were rinsed with cold 0·1 M S-collidine buffer and then fixed in cold 2% osmium tetroxide in 0·1 M S-collidine buffer for 45 min. The fixed cells were stained with 2% uranyl acetate in distilled water to enhance membrane and fibrillar structures for 60 min at room temperature. The cells were dehydrated with graded alcohol and embedded in Epon 812 in the usual way. Thin sections cut with a Porter-Blum MT-1 microtome (Ivan Sorvall Inc., Norwalk, Conn., U.S.A.) were stained with lead acetate, mounted on 150-mesh grids coated with collodion film and examined in a Hitachi HU-12A electron microscope (Hitachi Ltd, Tokyo, Japan). Measurements were with a magnifying measuring eyepiece on prints of known enlargement. Thick sections were also prepared for light microscopy and stained with toluidine blue.

### RESULTS

### *I. EM observation of AH136B cell adhesiveness without treatment with EDTA*

The suspension of AH136B cells (forming islands composed mostly of 10–30 cells), collected from the ascitic fluid withdrawn 10 days after i.p. inoculation of the cells, was kept at 37°C for 20 min and sedimented for EM study. In general, the external shape of the cell islands was round or oval, and the individual cells showed close contact (Fig. 1). The close contact in the apical portion of the cell islands was, as a rule, characterized by tight junctions (Fig. 2), while that in the inner portion consisted largely of simple apposition, and partly of intermediate junctions and desmosomes (Fig. 2). The mean number of tight junctions, desmosomes and intermediate junctions in the cell islands, when counted for 150 nuclei in cross-section by the method of Overton (1973), was approximately 100, 14 and 28 in that order. Desmosomes and intermediate junctions were apparently less frequent.

The tight junctions observed had a narrow gap of less than 4 nm which was formed by close approximation of outer leaflets and their punctate fusion, resembling that described by Trelstad, Hay and Revel (1967) (Fig. 3a). The regular distribution and constant presence of such tight junctions in adequately oriented sections suggested that they may form continuous belts around the cells,

FIG. 1.—EM picture of AH136B cell island. It is round and the individual cells adhere closely. ×2000.

as reported by Farquhar and Palade (1963). The desmosomes observed (*ibid.*) consisted of 2 outer leaflets running in a parallel fashion and separated by an intercellular space of about 16 nm, containing a central disc of electron-dense materials (Fig. 3b). In the cytoplasm subjacent to each inner leaflet, one distinct laminar plaque running parallel to the membranes was observed, and was accompanied by prominent endoplasmic fibrils (Fig. 3b). The intermediate junctions consisted of 2 outer leaflets disposed in a parallel fashion and separated by an intercellular space of less than 20 nm, exhibiting low electron density, and resembling those described by Farquhar and Palade (1963). In the cytoplasm subjacent to the inner leaflets, moderate electron density was revealed (Fig. 3c). The simple apposition observed was composed of apposed plasma membranes separated by a space of 10–30 nm showing no electron density, as was seen by Farquhar and Palade (1963). The structure consisted of 2 outer leaflets disposed in a parallel fashion, showing focal membrane undulation of varying degree (Fig. 3d).

*II. EM observation of AH7974 cell adhesiveness without treatment with EDTA*

The suspension of AH7974 cells (forming islands composed usually of less than

FIG. 2.—Higher magnification of part of Fig. 1. The individual cells show close contact of cell surface. Tight junctions (T) are found in the apical portion of the cell island. Simple apposition (S), intermediate junctions (I) and desmosomes (D) are observed in the inner portion. ×4600.

13 cells), collected from the ascitic fluid withdrawn 10 days after i.p. inoculation of the cells, was kept at 37°C for 20 min and then sedimented for EM examination. In general, the external shape of the cell islands was rather irregular, and the individual cells seemed to be in a relatively loose contact (Fig. 4). The close contact in the apical portion of the cell islands was characterized by tight junctions, as shown on AH136B cell islands. Although the areas of cellular apposition were smaller than those of AH136B cells, the areas showing close contact had many intermediate junctions and desmosomes, while simple apposition was apparently less frequent than that observed in AH136B cell islands. EM pictures of these binding structures observed in AH7974 cell islands were essentially the same as those in AH136B. It was thus suggested that AH7974 might be more convenient than AH136B for studying the structure of intermediate junctions and desmosomes in cell islands. When counted for 150 nuclei in cross-section, the mean numbers of tight junctions, desmosomes and intermediate junctions were about 100, 80 and 64, respectively.

*III. EM observation of AH7974 and AH136B cell-adhesiveness under treatment with EDTA*

When treated with 2 mM EDTA at 37°C for 20 min, tight junctions in the apical portion of AH136B cell islands remained unchanged, but apposed membranes of the cells in the inner portion showed a distinct separation (Fig. 5a). This apparent separation was assumed to develop from simple apposition, intermediate junctions and desmosomes (Fig. 5b), as seen before treatment with EDTA (Fig. 2). The mean number of tight junctions, desmosomes and intermediate junctions was approximately 100, 0 and

0 respectively per 150 nuclei in cross-section.

Although EM alteration by EDTA in desmosomes and intermediate junctions was not seen in AH136B cell islands because of the lower frequency of these structures (Fig. 2), changes were confirmed in AH7974 cell islands. A striking separation in the inner portion of the cell islands was revealed, but tight junctions remained intact (Fig. 6). The EM changes in desmosomes after EDTA seemed to occur in the following stages: (1) the central disc of electron-dense materials became obscure or disappeared, but other elements in the structure remained unchanged (Fig. 7a); (2) the central disc disappeared, intercellular space dilated to more than 30 nm and endoplasmic laminar plaque became obscure (Fig. 7b); and (3) widely dilated intercellular space developed with active formation of microvilli and disappearance of central disc and endoplasmic laminar

FIG. 3a.—Tight junction observed between adjacent AH136B cells. It is characterized by a narrow gap (G) of less than 4 nm, formed by close approximation and punctate fusion of outer leaflets (F). ×64,000.

FIG. 3b.—Desmosome observed in adjacent AH136B cells. Two outer leaflets are separated by about 16 nm, showing central disc of electron-dense materials. One electron-dense laminar plaque (P) adjacent to the inner leaflet is seen in the cytoplasm. Many endoplasmic fibrils (indicated by arrow) are related to the plaque. ×71,500.

FIG. 3c.—Intermediate junction observed in adjacent AH136B cells. Two outer leaflets are parallel and separated by 10–20 nm with low electron density. In the cytoplasm subjacent to the inner leaflet, electron-dense materials are seen. ×43,000.

FIG. 3d.—Simple apposition observed between adjacent AH136B cells. Two plasma membranes are parallel and separated by intercellular space of 10–30 nm showing no electron density. No specialized junctional structure. 45,000.

plaque (Fig. 7c). However, endoplasmic fibrils seemed to remain in an almost normal state. Unchanged desmosome structure was apparently less frequent. The morphological change in intermediate junctions after EDTA was dilation of intercellular space to more than 45 nm and decrease in the electron-dense materials in the cytoplasm subjacent to the inner leaflets (Fig. 7d). When counted for 150 nuclei in cross-section, the mean number of tight junctions, desmosomes and intermediate junctions with structures identical to those observed in untreated AH7974 cells was 100, 9 and 6, respectively.

### *IV. Reconstruction of binding structures by readministration of Ca ions*

AH136B cell islands, previously treated with 2 mM EDTA, were carefully washed with BSS, suspended in the same solution at 37°C for 80 min and then sedimented for EM examination.

After such treatment, in the inner portion of the cell islands, the close cell

FIG. 4.—EM picture of AH7974 cell island. The shape is rather irregular. The areas of cellular apposition are small, but many intermediate junctions (I) and desmosomes (D) can be found in the inner portion. Simple apposition (S) is less frequent. Tight junctions are observed in the apical portion. ×6700.

contact consisting largely of simple apposition and partly of intermediate junctions and desmosomes was observed (Fig. 8), suggesting a considerable reconstruction of the binding structures seen in AH136B cell islands before EDTA (Fig. 2). When counted for 150 nuclei in cross-section, the mean numbers of tight junctions, desmosomes and intermediate junctions which were recognisable, were about 100, 10 and 19, respectively. The number of tight junctions was the same before and after readministration of Ca ions.

AH7974 cell islands, previously treated with 2 mM EDTA, were similarly washed with BSS, suspended in the same solution at 37°C for 80 min and then sedimented. A considerable reconstruction of altered close contact of the cells in the inner portion of the cell islands was revealed (Fig. 9). This consisted of clearly defined desmosomes (Fig. 10a), intermediate junctions (Fig. 10b) and simple apposition (Fig. 10c). The mean numbers of tight junctions, desmosomes and intermediate junctions were about 100, 55 and 40 respectively for 150 nuclei in cross-section. The number of tight junctions was the same before and after readministration of Ca ions. In a control experiment using AH7974 cell islands untreated with EDTA, no morphological change in any of the binding structures in the apical and inner portions of the cell islands was found after incubation of the cells with BSS at 37°C for 20 or 100 min.

### DISCUSSION

The present findings show that both AH136B cells and AH7974 cells form islands in which the individual cells adhere by known binding structures, including simple apposition, intermediate junctions, desmosomes and tight junctions

(Figs. 2, 4). The close contact of the apical portion of the cell islands was, as a rule, composed of tight junctions with a narrow gap (Fig. 3a). The close contact of the inner portion of AH136B cell islands consisted largely of simple apposition (Fig. 3d) and partly of intermediate junctions (Fig. 3c) and desmosomes (Fig. 3b). On the other hand, the close contact of the inner portion

FIG. 5a.—AH136B cell island after treatment with 2 mM EDTA. The cells are separated by a wide intercellular opening with many cytoplasmic processes. Intact tight junctions (T) remain in the apical portion. ×6650.

FIG. 5b.—Marked separation of intercellular junctions (indicated by arrow) in the inner portion of AH136B cell island after EDTA. No change in the tight junction (T) in the apical portion. ×9600.

FIG. 6.—Striking separation (indicated by arrow) of intercellular junctions in the inner portion of AH7974 cell island after EDTA. Desmosomes (D) and intermediate junctions (I) clearly decreased in number. No change in tight junctions (T) in the apical portion. ×3800.

FIG. 7a.—Desmosome observed between adjacent AH7974 cells after EDTA. The structure shows an obscure central disc of electron-dense materials. However, other elements of the structure remain almost intact. ×52,000.

of AH7974 cell islands had many intermediate junctions and desmosomes (Fig. 4), simple apposition being less frequent.

The above difference in the binding manner suggests that AH136B cell islands are suitable for studying the alteration of simple apposition, and AH7974 cell islands for studying changes in intermediate junctions and desmosomes. Treatment with 2 mM EDTA induced a distinct separation of close contact in the inner portion of AH136B cells, while tight junctions remained intact (Figs. 5a, b), and AH7974 cells responded similarly (Fig. 6).

In a systematic investigation of the effects of proteolytic enzymes (trypsin), chelators (EDTA) and detergents (sodium desoxycholate, DOC) on desmosomes originating in a variety of tissues, Borysenko and Revel (1973) divided desmosomes into two broad categories: one

FIG. 7b.—Desmosome between adjacent AH7974 cells after EDTA. The structure has a wide intercellular space of more than 30 nm, no central disc of electron-dense materials, and obscure laminar plaque adjacent to the inner leaflet. Endoplasmic fibrils (arrows) remain almost unchanged. 52,000.

FIG. 7c.—Desmosome observed between adjacent AH7974 cells after EDTA. The structure has a distinct intercellular opening with many microvilli, no central disc of electron-dense materials nor laminar plaque adjacent to the inner leaflet. However, endoplasmic fibrils (arrows) remain almost intact. ×28,000.

FIG. 7d.—Intermediate junction observed between adjacent AH7974 cells after EDTA. The structure has a distinct intercellular opening of 45 nm and decreased electron-dense material adjacent to the inner leaflet. ×52,000.

FIG. 8.—Considerable reconstruction of altered binding structures in AH136B cells by readministration of Ca ions. The contact of individual cells resembles that in cells untreated with EDTA. T, tight junction; S, simple apposition; D, desmosome. ×4100.

group, sensitive to trypsin or DOC but insensitive to EDTA, is functionally stable in maintaining cell-to-cell contacts for long periods, as seen in stratified squamous and many glandular epithelia, and the other group, sensitive to EDTA but insensitive to trypsin or DOC, is physiologically labile or plastic, and allows intercellular passage of substances, as seen in simple columnar epithelia. Their findings that the extracellular components of desmosomes from simple columnar epithelia are EDTA-sensitive are of special interest, because alteration by EDTA of desmosomes in AH7974 cells was initiated by disappearance of the central disc of electron-dense materials (Fig. 7a). This suggests that desmosomes in AH7974 cells belong to the group sensitive to EDTA. The effect of EDTA on simple apposition, intermediate junctions and desmosomes in tumour cells was readily reversed in the absence of EDTA by Ca ions (Figs. 8, 9, 10a, b, c).

FIG. 9.—Considerable reconstruction of binding structures in AH7974 cells by readministration of Ca ions. There is an increase in desmosomes (D) and intermediate junctions (I). T, tight junction; S, simple apposition. ×4400.

FIG. 10a.—Desmosome between AH7974 cells reconstructed after readministration of Ca ions. ×44,400.

FIG. 10b.—Intermediate junction between AH7974 cells reconstructed after readministration of Ca ions. ×41,300.

FIG. 10c.—Simple apposition in AH7974 cells reconstructed after readministration of Ca ions. ×20,000.

The present results suggest that treatment with EDTA induces a decrease in Ca ions in tumour cell surfaces, resulting in a partial separation of tumour cells held together by simple apposition, intermediate junctions and desmosomes. Since tight junctions were not affected by EDTA, complete separation of tumour cells would need other conditions. Recent observations in our laboratory have demonstrated that a certain neutral protease isolated from AH109A cells can induce a complete separation of AH136B cells at a low activity, provided the cells were previously treated with EDTA. This neutral protease (Koono, Ushijima and Hayashi, 1974) induced no cellular damage *in vitro*. Similar separation of AH136B cells previously treated with EDTA, was provoked by a thermostable peptide from tumour tissue capable of releasing the neutral protease (Koono, Katsuya and Hayashi, 1974), suggesting that this neutral protease may disrupt tight junctions. Investigating the fine structure of freeze-cleaved tight junctions from epithelia of rat small intestine, Staehelin (1973) has postulated that the fragments of tight junctions can be internalized and broken down in lysosome-like vesicles. In this respect, it would be of interest to study whether the neutral protease may be isolated from lysosomes of AH136B or AH7974 cells.

We would like to record our appreciation to Dr H. Satoh at the Sasaki Institute, Tokyo, for a generous supply of rat ascites hepatoma AH7974 cells. This work was supported in part by a special grant for cancer research from the Japanese Ministry of Education, Science and Culture and by a grant from the Shionogi Pharmaceutical Company, Osaka, Japan.

REFERENCES

BORYSENKO, J. Z. & REVEL, J. P. (1973) Experimental Manipulation of Desmosome Structure. *Am. J. Anat.*, **137**, 403.

CARRUTHERS, C. & SUNTZEFF, V. (1944) The Role of Calcium in Carcinogenesis. *Science, N.Y.*, **99**, 245.

COMAN, D. R. (1944) Decreased Mutual Adhesiveness, a Property of Cells from Squamous Cell Carcinomas. *Cancer Res.*, **4**, 625.

DELONG, R. P., COMAN, D. R. & ZEIDMAN, I. (1950) The Significance of Low Calcium and High Potassium Content in Neoplastic Tissue. *Cancer, N.Y.*, **3**, 718.

FARQUHAR, M. G. & PALADE, G. E. (1963) Junctional Complexes in Various Epithelia. *J. Cell Biol.*, **17**, 375.

ISHIMARU, Y., ISHIHARA, H. & HAYASHI, H. (1975) An Electron Microscopic Study of Tumour Cell Adhesiveness Induced by Aggregation Factor from Rat Ascites Hepatoma Cells. *Br. J. Cancer*, **31**, 207.

KOONO, M., KATSUYA, H. & HAYASHI, H. (1974) Studies on the Mechanisms of Invasion in Cancer. IV. A Factor Associated with Release of Neutral Protease of Tumor Cell. *Int. J. Cancer*, **13**, 334.

KOONO, M., USHIJIMA, K. & HAYASHI, H. (1974) Studies on the Mechanisms of Invasion in Cancer. III. Purification of a Neutral Protease of Rat Ascites Hepatoma Cell Associated with Production of Chemotactic Factor for Cancer Cells. *Int. J. Cancer*, **13**, 105.

KUDO, K., TASAKI, I., HANAOKA, Y. & HAYASHI, H. (1974) A Tumour Cell Aggregation Promoting Substance from Rat Ascites Hepatoma Cells. *Br. J. Cancer*, **30**, 549.

MCCUTCHEON, M., COMAN, D. R. & MOORE, F. B. (1948) Studies on Invasiveness of Cancer: Adhesiveness of Malignant Cells in Various Human Adenocarcinomas. *Cancer, N.Y.*, **1**, 460.

OVERTON, J. (1973) Experimental Manipulation of Desmosome Formation. *J. Cell Biol.*, **56**, 636.

SEDAR, A. W. & FORTE, J. G. (1964) Effects of Calcium Depletion on the Junctional Complex Between Oxyntic Cells of Gastric Glands. *J. Cell Biol.*, **22**, 173.

STAEHELIN, L. A. (1973) Further Observations on the Fine Structure of Freeze-cleaved Tight Junctions. *J. Cell Sci.*, **13**, 763.

TRELSTAD, R. L., HAY, E. D. & REVEL, J. P. (1967) Cell Contact During Early Morphogenesis in the Chick Embryo. *Develop. Biol.*, **16**, 78.